# Pre and post-operative evaluation of gastroesophageal reflux and esophageal motility in neurologically impaired children using combined pH–multichannel intraluminal impedance measurements

**DOI:** 10.1007/s00383-013-3295-7

**Published:** 2013-03-22

**Authors:** Suguru Fukahori, Kimio Asagiri, Shinji Ishii, Yoshiaki Tanaka, Shin-ichiro Kojima, Nobuyuki Saikusa, Yoshinori Koga, Motomu Yoshida, Daisuke Masui, Naoko Komatsuzaki, Yoshitaka Seki, Minoru Yagi

**Affiliations:** 1 Department of Pediatric Surgery, Kurume University School of Medicine, 67 Asahi-machi, Kurume, Fukuoka 830-0011 Japan; 2 Department of Pediatrics, Kurume University School of Medicine, 67 Asahi-machi, Kurume, Fukuoka 830-0011 Japan

**Keywords:** Multichannel intraluminal impedance measurements, Gastroesophageal reflux, Neurologically impaired, Esophageal motility, Dry swallow

## Abstract

**Background:**

Gastroesophageal reflux disease (GERD) in patients with neurological impairment (NI) has not been fully studied before and after fundoplication procedure because their characteristics such as generalized gastrointestinal dysmotility, non-acid reflux, and the proximal reflux due to feeding of enteral nutrition via a nasogastric tube prevent their GERD from being detected by 24 h pH monitoring. The aim of this study was to elucidate whether multichannel impedance–pH measurement (pH/MII) is able to detect the subtypes of GERD and the differences in the reflux episodes of the severity of GERD, the ingestion pathway, and before and after fundoplication. The second aim was to determine whether a trial evaluation of dry swallows was able to be used to assess the esophageal motility of NI patients as an alternative examination.

**Patients and methods:**

The 24 h pH/MII was conducted on 20 NI children [15 were the patients before Nissen’s fundoplication (BN), of whom, six were fed orally (FO) and nine were fed via nasogastric tube (NGT), and five were the patients after Nissen’s fundoplication (AN)]. All reflux episodes were evaluated and compared between patients with pathological GERD (PG) and non-pathological GERD (NG) and between patients who had FO and NGT and patients between BN and AN. Dry swallows were conducted to evaluate the esophageal motility. The average bolus presence time (BPT) and total bolus transit time (TBTT) were compared between the PG and NG, FO and NGT, and the BN and AN subgroups.

**Results:**

A total of 1,064 reflux episodes were detected by pH/MII. Of those, 303 (28.5 %) were non-acid-related and 477 episodes reached the proximal esophagus. Of the 12 patients (57.1 %) showing pathological GERD, two cases (16.7 %) demonstrated predominantly weakly acidic PG. More than half of the reflux episodes of PG patients reached to the proximal esophagus. The numbers of total reflux and proximal reflux episodes in the PG were significantly higher than those in NG patients. The number of proximal reflux episodes in the FO group was significantly higher than that in the NGT groups, whereas NGT patients showed more non-acidic reflux episodes than FO patients. A trial evaluation of dry swallows demonstrated no significant differences in this study.

**Conclusion:**

The pH/MII was useful to detect the subtype of GERD in NI patients which could not be detected by 24 h pH monitoring. It can, therefore, be considered to have first priority for testing NI patients who are suspected to be suffering from GERD.

## Introduction

The high incidence of gastroesophageal reflux disease (GERD) in neurological impairment (NI) patients is well recognized [1]. In neurologically normal adults and children, most reflux episodes occur during transient lower esophageal sphincter relaxation (TLESR) [2]. On the other hand, in NI patients, along with TLESR, abnormal modulation of the extrinsic innervation due to a damaged central nervous system (CNS) or hypoxic-ischemic damage to the enteric nerves causes abnormal esophageal motility [3]. Consequently, generalized gastrointestinal dysmotility generates neuromuscular incoordination of the esophagus and impairs the LES mechanism, which has been considered to be the main cause of GERD in NI patients. Moreover, a combination of several factors such as scoliosis, a horizontal position, constipation, muscular tone disorder, and seizures are also known to aggravate GERD in NI patients [4]. Delayed gastric emptying has also been recognized as one of the causes of GERD [5]. Thus, GERD in NI children is considered to be caused by several factors [6], which means that severity of the GERD depends on the individual patient’s condition. Further complicating the situation, NI patients are not able to complain of GERD-related symptoms, so detecting GERD in NI patients is often difficult. Many NI patients who have not received any GERD examinations or treatments might also be suffering from GERD [7]. Furthermore, NI children are generally fed via a nasogastric tube with enteral nutrition; therefore, the acid reflux would be neutralized by the feeding, and it is considered that they would be more likely to be suffering from non-acid reflux episodes via the nasogastric tube.

The surgical management of GERD such as fundoplication has become a standard procedure in NI patients [8]. The majority of the previous studies have advocated that the fundoplication procedure controls reflux by increasing the basal lower esophageal sphincter (LES) [9]. An effective mechanical anti-reflux barrier can be created by fundoplication, although the underlying dysmotility remains. Nevertheless, post-fundoplication problems are more common in NI patients than normal subjects. Several follow-up studies have shown a high incidence of complication and recurrent reflux after fundoplication procedures in NI patients [10]. If a subtype of GERD which does not require anti-reflux surgery can be detected, it might be possible to reduce the number of NI patients suffering from post-fundoplication complications.

The 24 h pH monitoring has been widely used as a gold standard method to evaluate GERD. However, non-acidic refluxes are not able to be detected by this conventional examination. pH/MII have been established as a pH independent measurement tool. The advantage of pH/MII is that it allows for the analysis of the movement, direction, and height attained by the bolus, making it possible to distinguish antegrade and retrograde bolus movement. Using an impedance catheter with integrated pH sensors, the pH of the reflux episodes can be determined simultaneously.

Meanwhile, to assess the esophageal motility, esophageal manometry studies using either a liquid or a viscous material have been the gold standard method and several reports have described their utility [11]. Manometry provides information about the esophageal pressure pattern and sphincter function, but does not provide information about the bolus transit. Fluoroscopy and scintigraphy have been used as alternative tool, though the applicability of these tools is limited in children due to the exposure to radiation.

The pH/MII was introduced to evaluate esophageal bolus transport and can provide information about the functional outcome of the esophageal motor function. Esophageal bolus clearance can be assessed by measurement of the BPT or TBTT and by classifying swallows as complete bolus transit (if bolus entry occurs at the most proximal site and bolus exit points are recorded in all three distal recording segments) or as incomplete bolus transit (if bolus exit is not identified at any of the three distal recording segments) [12]. Validation studies have found an excellent correlation between the pH/MII and videofluoroscopy. There is also a good correlation between the pH/MII and manometry in healthy subjects and in patients with GERD [13]. However, NI patients usually have difficulty in swallowing and a high-risk of aspiration, which means that an evaluation via liquid or viscous swallows and measuring the percentage of complete bolus transit would be unsuitable for them. Therefore, the present study tried to evaluate dry swallows, although it has been reported that dry swallows are inferior to liquid or viscous swallows in esophageal manometry studies [11]. Moreover, in NI children, the serpentine esophagus that is present due to scoliosis often makes it difficut to insert a catheter. It is also often difficult to conduct multiple examinations to assess NI patients with severe swallowing disorders for GERD. For these reasons, pH/MII should be ideal for NI patients, and a better therapeutic strategy for GERD in NI patients should be established based on an objective assessment that can assess all reflux episodes.

The aim of this study was to elucidate whether multichannel impedance–pH measurement (pH/MII) is able to detect the subtypes of GERD and the differences in the reflux episodes of the severity of GERD, the ingestion pathway, and before and after fundoplication. The second aim was to determine whether a trial evaluation of dry swallows was able to be used to assess the esophageal motility of NI patients as an alternative examination.

## Patients and methods

The study included 20 NI patients, 6 males and 14 females, aged 1–21 years. Fifteen patients were referred due to symptoms suggesting GERD and five had already undergone a surgical procedure. Six patients were being fed orally (FO) and nine were being fed via a nasogastric tube (NGT), five patients had undergone Nissen’s fundoplication (PN). The medication for GERD was stopped at least 3 days before the subjects entered the study. The study protocol was approved by the Kurume University Ethical Committee (No. 2575). Informed consent was obtained from the families before starting this study.

A multiple intraluminal impedance catheter (outer diameter, 2 mm) with two pH antimony electrodes and seven impedance electrodes (Sandhill Scientific, Inc, Highlands Ranch, CO, USA) was used. The catheter was inserted transnasally through the esophagus, and the pH sensor placement was confirmed by radiography. The impedance data were automatically evaluated using the BioVIEW analysis software program and each tracing was manually reviewed by the same author.

Liquid reflux was defined by pH/MII as when a fall in impedance ≥50 % from baseline occurred in at least two consecutive channels in an aboral direction.

Each type of reflux was defined as follows: acidic reflux was diagnosed in case of associated pH drop to ≤4, weakly acidic reflux was diagnosed in cases associated with a pH value between 4 and 7, and weakly alkaline reflux was diagnosed in cases associated with a pH above 7. The pH reflux index was defined as the percentage of time with a pH ≤4. We defined 4.2 % as the upper cut-off value. The bolus exposure index was defined as the percentage of time with retrograde movement of intraluminal esophageal material. We defined 1.4 % as the upper cut-off value (higher than the 95th percentile of normal 24 h MII values, as suggested by Shay et al. [14] in an adult series of healthy patients).

Pathological GERD was defined as cases where the pH reflux index exceeded 4.2 % or the bolus exposure index exceeded 1.4 %.

According to the above definitions, all patients were evaluated and diagnosed to have PG or NG.

All reflux episodes were evaluated and compared between the PG and NG groups (five patients who received surgical procedures were excluded.), between the FO and NGT groups, and between the BN and AN groups.

Esophageal function was assessed by manual evaluation of two specific motility parameters: the bolus presence time (BPT) (the time elapsed between bolus entry and bolus exit at each impedance measurement site) and the total bolus transit time (TBTT) (the time elapsed between bolus entry at the most proximal recording segment and bolus exit at the most distal recording segment). Dry swallows, which decrease in impedance to 50 % of the baseline value in all recording channels, with downward direction without a prandial period, were conducted to evaluate the esophageal motility. Five complete dry bolus transits were evaluated. The average BPT and TBTT were calculated between PG and NG patients (five patients who received surgical procedures were excluded), between the FO and NGT patients, or between the BN and AN patients.

All statistical analyses were performed with the StatMate III software program (ATMS Co, Ltd, Tokyo, Japan). The Mann–Whitney *U* test was used for the nonparametric analyses. Values of *p* < 0.05 were considered to be statistically significant.

## Results

When we evaluate the number of reflux episodes (Table 1), a total of 1,064 episodes were detected by MII during the study period. Of those, 761 episodes were acidic, whereas 303 were non-acidic. A total of 477 episodes reached the proximal esophagus. Eleven patients (60 %) were diagnosed to have PG, of these, two cases (16.7 %) demonstrated predominantly weakly acidic PG.

**Table 1:** The summary of the number of reflux episodes

	Total	Acid	Weakly acidic	Weakly alkaline
All cases (*n* = 20)				
T	1,064 (53.2)	761 (38.1)	296 (14.8)	7 (0.3)
P	477 (23.9)	355 (17.8)	122 (6.1)	0 (0.0)
PG (*n* = 11)				
T	660 (60.0)	476 (43.3)	182 (16.5)	2 (0.2)
P	388 (35.3)	281 (25.5)	107 (9.7)	0 (0.0)
NG (*n* = 4)				
T	128 (32.0)	80 (20.0)	45 (11.3)	3 (0.7)
P	27 (6.8)	15 (3.8)	12 (3.0)	0 (0.0)
FO (*n* = 6)				
T	417 (69.5)	311 (51.9)	104 (17.3)	2 (0.3)
P	263 (43.8)	204 (34.0)	59 (9.8)	0 (0.0)
NGT (*n* = 9)				
T	371 (41.2)	245 (27.2)	123 (13.7)	3 (0.3)
P	152 (16.9)	92 (10.2)	60 (6.7)	0 (0.0)
BN (*n* = 15)				
T	788 (52.5)	556 (37.1)	227 (15.1)	5 (0.3)
P	415 (27.7)	296 (19.8)	119 (7.9)	0 (0.0)
AN (*n* = 5)				
T	278 (55.6)	205 (41.0)	69 (13.8)	4 (0.8)
P	75 (15.0)	60 (12.0)	15 (3.0)	0 (0.0)

() Mean value

*T* Total number of reflux episodes, *P* Number of proximal reflux episodes, *PG* Pathological GERD, *NG* Non-pathological GERD, *FO* Fed orally, *NGT* Fed via nasogastric tube, *BN* Before Nissens fundoplication, *AN* After Nissens fundoplication

**Fig. 1:**
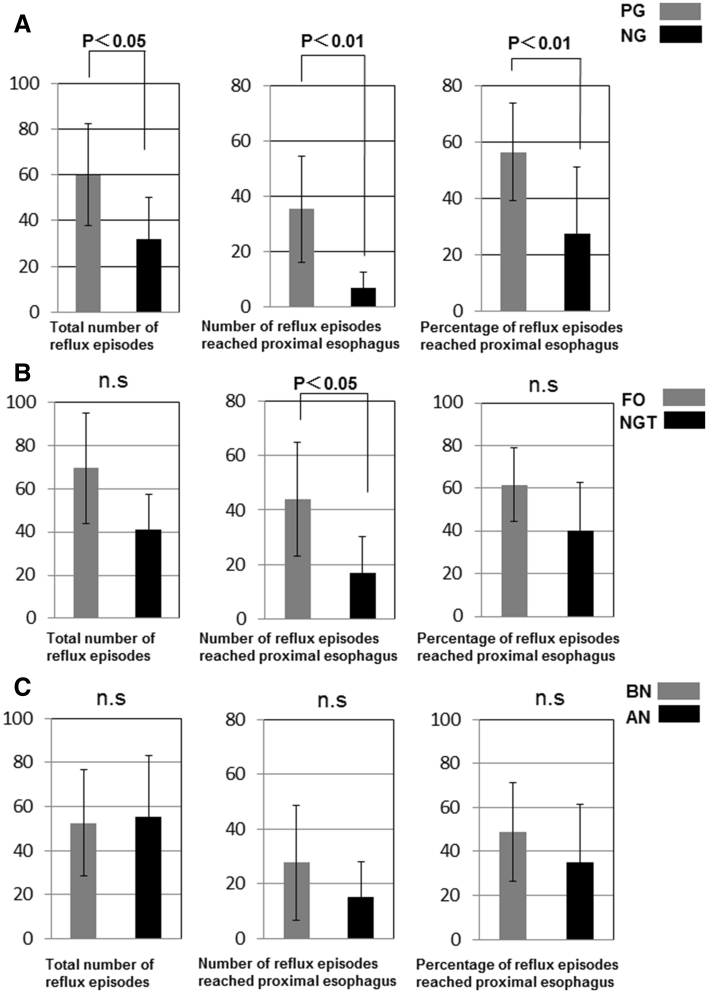
**a** The number of total reflux and proximal reflux episodes in PG patients was significantly higher than that in NG patients (60.8 vs. 38.9 and 56.1 vs. 28.4, respectively). **b** The total number of reflux episodes that reached the proximal esophagus in the FO group was significantly higher than that in the NGT and PN groups. **c** There was no significant difference between the BN and AN groups

In the PG group (*n* = 11), there were a total of 660 reflux episodes; of these, 476 were acidic and 184 were non-acidic. Slightly more than half (388) of these reflux episodes reached the proximal esophagus. In the NG group (*n* = 4), there was a total of 128 reflux episodes, 80 of which were acid and 48 of which were non-acidic. Twenty-seven of these reflux episodes reached the proximal esophagus in these patients. The numbers of total reflux episodes and proximal reflux episodes in PG patients were significantly higher than those in NG patients (60.0 vs. 32.0 and 35.3 vs. 6.8, respectively) (*p* < 0.05) (Fig. 1a).

When we compared the FO and NGT groups, there was a total of 417 reflux episodes in the FO group (*n* = 6). Of these, 311 were acidic and 106 were non-acidic. There were 263 reflux episodes that reached the proximal esophagus. In the NGT group (*n* = 9), there was a total of 371 reflux episodes. Of these, 245 were acidic and 126 were non-acidic. The total number of reflux episodes that reached the proximal esophagus was 152. The total number of proximal reflux episodes in the FO group was significantly higher than that in the NGT and PN groups (43.8 vs.16.9) (*p* < 0.05) (Fig. 1b).

Comparing the BN and AN groups, there was a total of 788 reflux episodes in the BN group (*n* = 15). Of those, 556 were acidic and 232 were non-acidic. There were 415 reflux episodes that reached to the proximal esophagus. In the AN group (*n* = 5), the total number of reflux episodes was 278. Of these, 205 were acidic and 73 were non-acidic. Seventy-five total reflux episodes reached the proximal esophagus. There were no significant differences between the two.

**Fig. 2:**
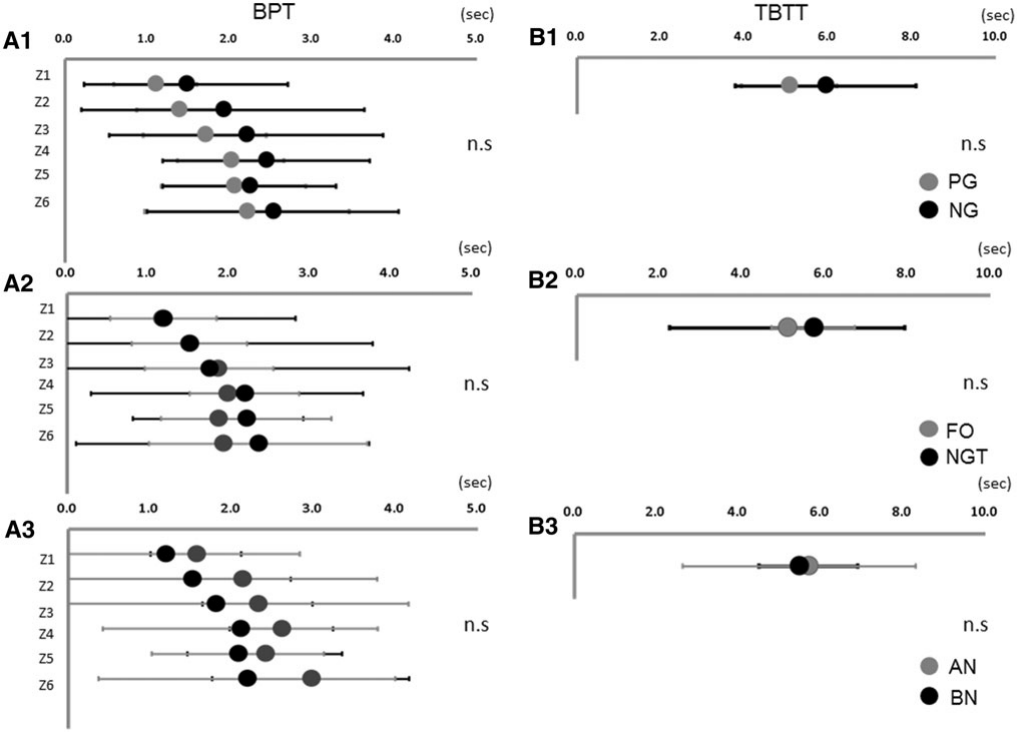
There were no significant differences in the BPT and TBTT between PG patients and NG patients (**a1** and **b1**), between FO and NGT (**a2** and **b2**), and between BN and AN patients (**a3** and **b3**)

Upon evaluating dry swallows, the average BPT (s) of all patients was 1.3 at Z1, 1.7 at Z2, 1.9 at Z3, 2.2 at Z4, 2.2 at Z5, and 2.4 at Z6. The average TBTT (s) was 5.52. In the PG group, the average BPT was 1.2 at Z1, 1.4 at Z2, 1.8 at Z3, 2.1 at Z4, 2.1 at Z5, and 2.3 at Z6. The average TBTT was 5.20. In the NG group, the average BPT was 1.3 at Z1, 1.7 at Z2, 1.9 at Z3, 2.2 at Z4, 2.0 at Z5, and 1.9 at Z6. The average TBTT was 6.2. There were no significant differences in the BPT **(**Fig. 2a1**)** and TBTT (Fig. 2b1) between these two groups.

When we compared between the FO and NGT groups, the average BPT of the FO group was 1.2 at Z1, 1.5 at Z2, 1.9 at Z3, 2.0 at Z4, 1.9 at Z5, and 1.9 at Z6. The average TBTT was 5.09. In the NGT group, the average BPT was 1.2 at Z1, 1.5 at Z2, 1.8 at Z3, 2.2 at Z4, 2.2 at Z5, and 2.4 at Z6. The average TBTT was 5.71. The average TBTT was 5.69. No significant difference was observed in the average BPT (Fig. 2a2) or TBTT (Fig. 2b2) between those two groups.

Comparing the BN and AN groups, the average BPT of the BN group was 1.2 at Z1, 1.5 at Z2, 1.8 at Z3, 2.1 at Z4, 2.1 at Z5, and 2.2 at Z6. In the AN group, the average BPT was 1.6 at Z1, 2.1 at Z2, 2.3 at Z3, 2.6 at Z4, 2.4 at Z5, and 3.0 at Z6. The average TBTT was 5.46. There were also no significant differences in the BPT (Fig. 2a3) and TBTT (Fig. 2b3) between these two groups.

## Discussion

Neurological impairment patients account for the great majority of GERD patients requiring anti-reflux surgery in the pediatric surgical field, but there have not been many studies that have evaluated the GERD of NI patients via an objective assessment, although there have been many reports about the effectiveness of anti-reflux procedures [15].

The present study was the first to conduct 24 h pH/MII for pediatric NI patients and evaluated dry swallows to assess their esophageal motility. The advantage of pH/MII is that once a catheter was inserted into the esophagus, we were able to recognize what occurred in the esophagus for 24 h by analyzing a combination of wave forms and the pH, which allowed us to assess the GERD as well as the esophageal motility, without requiring the insertion of another catheter.

There has so far been only one report that has evaluated GERD in NI children [7] and no previous study has so far conducted 24 h pH/MII for NI patients. In the previous study, Del Buono et al. [7] reported that in 16 NI children with 12-h impedance recordings, more than half of the reflux events were non-acidic and would have gone undetected by conventional pH measurement. In the present study, non-acid reflux accounted for only 28.5 % of episodes, though the NGT group showed a higher percentage of non-acidic reflux (33.2 %) than FO groups. The differences in the results between the previous studies and our present study might have been due to the differences in the age of patients and the duration of the study. In addition, two cases (16.7 %) demonstrated PG dominated by weakly acidic reflux, which would not have been detected by conventional pH monitoring, and has not been mentioned by previous reports.

When we evaluated the number of reflux episodes in the present study, the patients with PG demonstrated more reflux episodes. This result was similar to those of previous studies in neurologically normal adults that indicated that the patients with erosive esophagitis or non-erosive esophagitis demonstrated a higher incidence of both acidic and non-acidic reflux episodes compared with healthy volunteers [16]. Another result that more than half of the reflux episodes reached the proximal esophagus (58.8 %) might be a new finding. This finding implies that NI patients with PG might have higher risk of aspiration than neurologically normal patients. Between the FO and NGT patients, the FO patients showed more proximal reflux episodes than NGT patients. Although some reports indicated that placing a nasogastric tube into the stomach increased the chance of reflux [17], this condition did not seem to affect the number of reflux episodes in the present study. Presumably, FO patients might have frequent reflux episodes due to TLESR. In contrast, no significant difference was observed in the number of reflux episodes between BN and AN patients. This result might be due to a comparison between different patients. As a result, further study is required between BN and AN in same NI patient.

There has been no previous report evaluating dry swallows by pH/MII. On the other hand, there has so far been only one report that evaluated the normal values for pH/MII parameter for liquid swallows in neurologically normal children and that report described that the values of pH/MII parameter were similar to those obtained in healthy adults [18]. One neurologically normal adult study tried to evaluate the esophageal motility of post-fundoplication patients by measuring the percentage of complete bolus transit using ten liquid and viscous swallows. It was found that the patients complaining of dysphagia or showing abnormal anatomy, such as the post-operative herniation of the stomach or a hiatal stricture, were more likely to have impaired esophageal clearance [19].

A trial evaluation of dry swallows by pH/MII in this study showed that there was no statistically significant difference in this study. However, considering the mechanism of GERD in NI patients, it is likely that a certain number of patients with factors associated with GERD or with post-fundoplication complications, such as intrathoracic herniation, might suffer from severe esophageal dysmotility. In the present study, the AN patients did not appear to suffer from any post-fundoplication complications. Further studies including NI patients with these complication could elucidate the effectiveness of the dry swallow studies via pH/MII.

To summarize, several subgroups of NI patients demonstrated characteristic GERD findings: a certain proportion of NI patients demonstrated predominantly weakly acidic PG. More than half of the reflux episodes of PG patients reached to the proximal esophagus. When PG and NG patients were compared, the PG patients showed both more reflux episodes and more proximal reflux episodes. When the FO and NGT patients were compared, it was found that FO patients suffered from more proximal reflux episodes, whereas NGT patients showed more non-acidic reflux episodes than FO patients. On the other hand, a trial evaluation of dry swallows detected no significant differences in this study.

Neverthless, the present study provided only limited information about the pathophysiology of GERD and esophageal motility in NI patients due to the small number of subjects included in the study and the variability of the patients’ ages. pH/MII still has its limitations due to the lack of normal values for children, the expensive cost of consumables, and the time required for the analysis.

In conclusion, pH/MII was useful to detect the subtype of GERD which could not to be detected by 24 h pH monitoring and can, therefore, be considered to have first priority for testing NI patients who are suspected to be suffering from GERD or post-fundoplication complications. A larger study will be required to further elucidate the pathophysiological mechanisms of GERD in NI patients.
